# Effect of MMP-2 gene silencing on radiation-induced DNA damage in human normal dermal fibroblasts and breast cancer cells

**DOI:** 10.1186/s41021-019-0131-x

**Published:** 2019-07-22

**Authors:** Gugalavath Shailender, Seema Kumari, Patnala Kiranmayi, Rama Rao Malla

**Affiliations:** 1 Cancer Biology Lab, Department of Biochemistry and Bioinformatics, Institute of Science, GITAM (Deemed to be University), Visakhapatnam, Andhra Pradesh India; 2 Department of Biotechnology, Institute of Science, GITAM Deemed to be University, Visakhapatnam, Andhra Pradesh India

**Keywords:** Ionizing radiation, Matrix metalloproteinase, DNA damage, Breast cancer, Fibroblasts

## Abstract

**Introduction:**

Diagnostic and therapeutic ionizing radiation (IR) is one of the well known long term risk factors of breast cancer. Extremely lethal consequences of IR causes double-strand breaks, which are mainly responsible for genomic instability, altered gene expression, and cell death.

**Findings:**

This study evaluated the effect of matrix metalloproteinases-2 (MMP-2) gene silencing using MMP-2 shRNA expression plasmids (pMMP-2) on IR induced cytotoxicity and DNA damage by MTT, dead green, γH2AX and comet assays in human normal dermal fibroblasts (HDFs) and MCF-7 human breast cancer cells. IR has decreased the viability of HDFs and MCF-7 cells with increasing IR (2-10Gy). IR induced DNA damage in both HDFs and MCF-7 cells. However, pMMP-2 transfection has increased the viability of irradiated HDFs (10Gy) and significantly decreased the viability of irradiated MCF-7 cells (10Gy). Further, DNA damage in terms of γH2AX foci decreased with pMMP-2 transfection in irradiated HDFs (10Gy) and increased in irradiated MCF-7 cells (10Gy). In addition, MMP-2 gene silencing using pMMP-2 decreased comet tail length in irradiated HDFs but increased in irradiated MCF-7 cells.

**Conclusions:**

The results conclude that pMMP-2 has protected HDFs and sensitized the MCF-7 cells from IR induced DNA damage. This differential response might be due to IR induced MMP-2 distinctive ROS generation in HDFs and MCF-7 cells.

**Electronic supplementary material:**

The online version of this article (10.1186/s41021-019-0131-x) contains supplementary material, which is available to authorized users.

## Introduction

The exposure of humans to ionizing radiation (IR) induces several types of genetic and somatic mutations leading to several types of cancers including breast cancer [1]. However, a single dose or fractionated dose of radiation has severe effects on the adjacent normal tissue during radiotherapy [2]. The high dose of radiation induces early and late skin effects and secondary neoplasm during radiotherapy of breast cancer [3].

Normal or cancer tissue is composed of extracellular matrix (ECM) and cellular constituents mainly fibroblasts. Fibroblasts have a major role in structural integrity, tissue repair, and deposition of the ECM and regulation of epithelial cell differentiation, inflammation and wound healing [4]*.* However, cancer-associated fibroblasts have a major role in proliferation, invasion, tumor progression, metastasis, and angiogenesis. Various studies have highlighted that dermal fibroblasts are pertinent and condemnatory target cells for IR [5]. Therefore, the development of novel and competent non-toxic radioprotectors is of great interest against the radiation-induced damages. Radioprotectors are important to safeguard the normal tissues during radiotherapy of breast cancer [6]. The naturally occurring thiol- and sulphur- containing compounds, pharmacological agents and phytochemicals present radioprotection, but non-specific, and showed side effects like cephalalgia, nausea, sickness and vomiting [7, 8].

Matrix metalloproteinases are a broad family of Zn containing proteases including collagenases, gelatinases, stromelysins, elastases and membrane-type [9]. Matrix metalloproteinases-2 (MMP-2) is a member of gelatinase sub-family plays a crucial role in ECM turnover. It degenerates the component of the basement membrane, alters the interstitial collagens I and III, native collagen IV. Previous reports have described that IR increased the MMP-2 activity in human bronchial epithelial cells [10], rat astrocytes [11], rat mesangial cells, rat kidney tubule epithelial cells [12], and human cultured fibroblasts [13]. Further, increased synthesis and activity of MMP-2 augment the biological aggressiveness of breast cancer [14]. MMP-2 activity was targeted using various phytochemicals [15]. But these phytochemicals are neither specific to cancer cells nor normal cells. However, RNAi mediated gene silencing protected normal cells [16–19]. The aim of the present investigation is to study the effect of MMP-2 gene silencing by transfection of MMP-2 shRNA expression plasmids (pMMP-2on radiation-induced DNA damage in human normal dermal fibroblasts (HDFs) and MCF-7 cells.

## Methods

### Chemicals and reagents

Dulbecco’s modified Eagle’s medium (DMEM), Fetal bovine serum (FBS), MTT reagent, Dead green viability stain, γH2AX primary antibody, Alexa Fluor-555 conjugated secondary antibody, Lipofectamine® 3000 Reagent were procured from Invitrogen, USA. Vista green DNA dye and MMP-2 primary antibody was procured from Cell Biolabs, USA and Santa Cruz Biotechnology respectively.

### Cell culture and maintenance

Human normal dermal fibroblast cells (HDFs) were obtained from the cell bank, Hi-Media, Mumbai; human breast cancer cells (MCF-7) were obtained from NCCS Pune. HDFs and MCF-7 cells were cultured in DMEM medium supplemented with 10% FBS and 100 U/mL gentamicin and maintained at 37^O^ C in 5% CO_2_ humidified environment.

### Construction of MMP-2 shRNA expression plasmids (pMMP-2)

The potential target sequences, from + 574, + 1035, + 1380 and + 1526 of MMP-2 (Genbank accession No. NM_001302510.1) were identified by scanning MMP-2 mRNA (cds) obtained from NCBI. Finally, one set of target sequence was obtained based on homology to human MMP-2. Web based design tool was used to design MMP-2shRNA with 21 complementary base pairs and with a 5′- TCAAGAG − 3′ loop sequence at centre and 5′ end with BamH1 and 3′-end with HindIII restriction site (https://www.invivogen.com/sirnawizard) (Table 1). Plasmid with scrambled sequence or non specific to MMP-2 gene (pSV-sh) was served as negative control. Commercially synthesized oligonucleotides of MMP-2shRNA where annealed and ligated with T4 DNA ligase into pSilencer2.0-U6vector (Ambion). Further, transformed into *E.coli* dh5α competent cells and plasmid was isolated using Qiagen Kit.

**Table 1:** Forward and reverse primers used for construction of pMMP-2sh vector

S.No	Gene	Forward Primer	Reverse Primer
1	MMP-2	5’GATCCGTACCTCGAGACAAATTCTGGAGATACATCAAGAGTGTATCTCCAGAATTTGTCTCTTTTTGGAAA3’	3’CATGGAGCTCTGTTTAAGACCTCTATGTAGTTCTCACATAGAGGTCTTAAACAGAGAAAAACCTTTTCGA5’

### Western blotting analysis for transfection efficiency

HDFs and MCF-7 cells were transfected with 2 μg of pMMP-2 or pSV using Lipofectamine 3000 Reagent in 1.5:1 ratio for 48 h. Whole cell proteins were extracted using RIPA buffer, concentration of protein were quantified and protein separation was performed by SDS PAGE to the extracted denatured protein. Separated protein in the PAGE where fetched by PVDF membrane by wet transfer method. To the blot MMP-2 primary antibody was added and incubated for 30 min further with secondary antibody specific to MMP-2 primary antibody. The blots were developed and image J software was used to quantify the density of bands. The blots were re-probed with GAPDH antibody, which served as loading control.

### Irradiation source

Cells were seeded in a 96-microwell plate and allowed to adhere overnight. A Mini X-ray tube (Amptek, Bedford, MA, USA) was used to produce x-rays with silver anode operated at 40 kV and 100 μA. Target was focused using brass collimator (2 mm diameter pinhole). After exposure to different dosages of IR (2Gy/30s), a monolayer of cells were transfected with or without 2 μg of pMMP-2 using Lipofectamine 3000 Reagent in 1.5:1 ratio and incubated for 48 h.

### MTT assay

Briefly, cells (5 × 10^3^/well) were seeded in a 96-well plate and allowed to adhere overnight. Initially, cells were irradiated with a different dose of IR at 2, 4, 6, 8 and 10Gy to determine the single effective dose at 24, 48 and 72 h. Further, at a single effective dose i.e., 10Gy cells were treated with or without 2 μg of pMMP-2 and incubated for 48. After incubation, MTT reagent (10 μl) was added and incubated for 2 h. Purple formazan crystals were dissolved in DMSO (100 μl) and absorbance was taken at 590 nm using ELISA plate reader (Bio-Rad, USA) [19].

### Dead green assay

Cells (1x10^4^cells/well) were irradiated with IR at 10Gy and further transfected with or without 2 μg of pMMP-2 for 48 h, fixed with paraformaldehyde (4%) and permeabilized with Triton-X 100(0.1%). Then 50 μL of dead green viability stain was added to each well and incubated for 30 min as per the manufacturer’s instructions. Stained cells were visualized under a fluorescent microscope (Lynx Microscope, USA), at an excitation (Ex) wavelength of 488 nm and an emission (Em) wavelength of 515 nm and images were captured at 20x resolution.

### Immunofluorescence assay of γH2AX

γH2AX expression was determined using the immunofluorescence assay. The cells were irradiated with IR (10Gy) further transfected with or without 2 μg of pMMP-2 and incubated for 48 h. Cells were fixed with 4% paraformaldehyde and permeabilized. Then cells were incubated with γH2AX mouse monoclonal antibody followed by anti-mouse Alexa Fluor 555 goat antibody. Fluorescent microscopy was used to visualize and capture image [19].

### Alkaline comet assay

The cells were irradiated with IR at 10Gy and further transfected with or without 2 μg of pMMP-2 and incubated for 48 h. Collected cells were suspended in PBS. Subsequently, cell sample and 1% of low melting agarose were diluted in 1:10 ratio, coated to comet slide in a volume about 100-120ul allowed to incubate at 37 °C. Then the slide was plated in a lysis buffer at 4 °C for 60 min followed by incubation in pre-chilled alkaline solution for 30 min. Electrophoresis was carried out by using the alkaline electrophoresis solution at 25 V and 300 mA for 20 min. The slide was washed with distilled water and incubated with 100ul of Vista green DNA dye at room temperature for 15 min. The images were captured by fluorescent microscopy. Length of the comet was determined using CASP Lab software [20].

## Results

### Transfection efficacy of pMMP-2 in HDFs and MCF-7 cells

To evaluate the efficacy of MMP-2 shRNA construct, the monolayer of HDFs and MCF-7 cells were transiently transfected with MMP-2 shRNA for 48 h and expression was analyzed by western blotting analysis. Western blotting analysis reveal that 3.5 fold decreased expression of the MMP-2 protein compared to pSV-sh in HDFs (Additional file 1 Figure S1a). Whereas in MCF-7 cells transfected with pMMP-2 decreased 3 fold compared to pSV-sh (Additional file 1 Figure S1b).

### Effect of MMP-2 gene silencing on radiation-induced DNA damage in HDFs

**Fig. 1:**
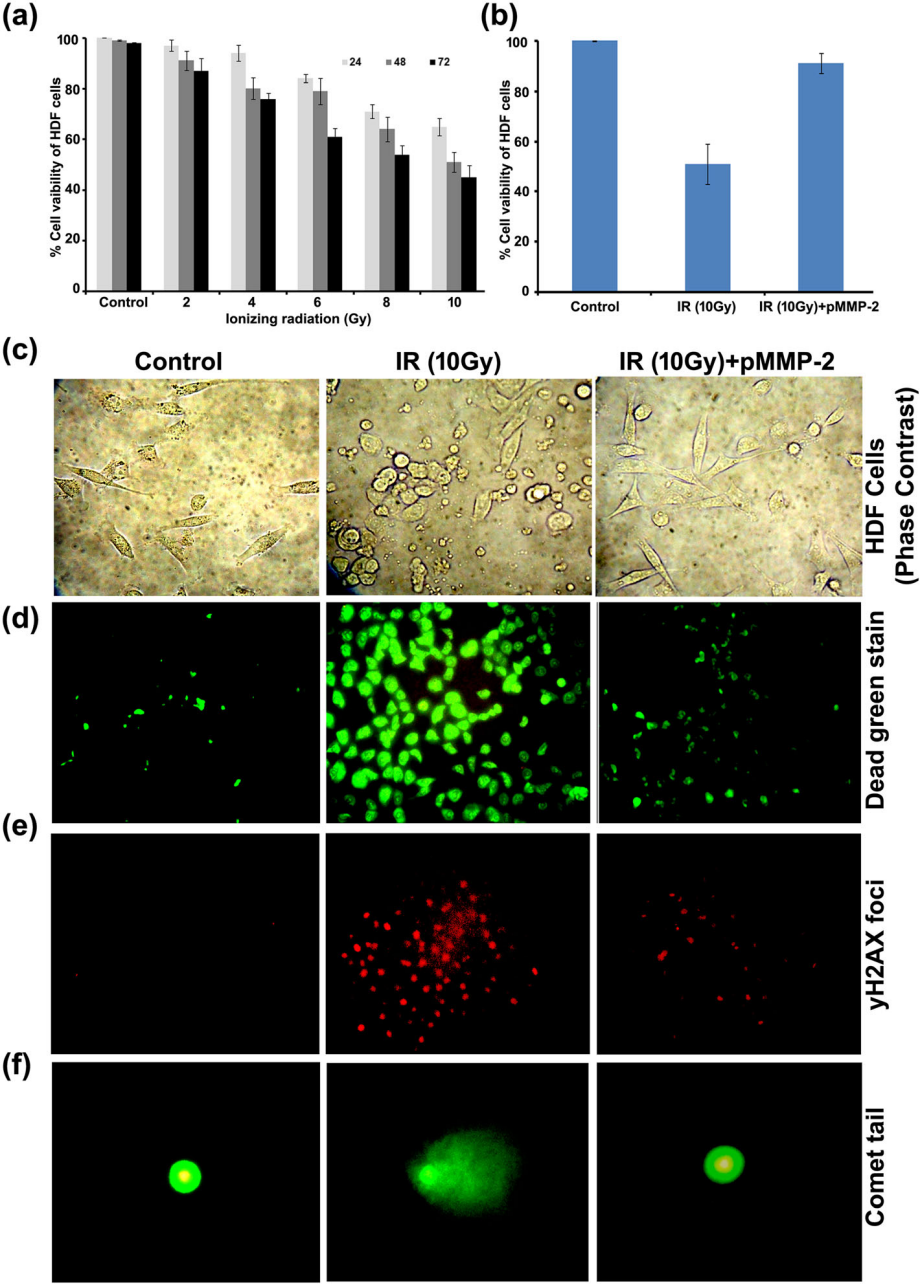
Effect of MMP-2 gene silencing in pMMP-2 transfected irradiated HDF cells. **a** Graphical representation of viability of HDF cells on irradiation (2-10Gy) analyzed by MTT assay. **b** Graphical representation of viability in pMMP-2 transfected HDF cells at single large dose i.e. 10Gy analyzed by MTT assay. **c** Morphological characterization under phase contrast microscopy of HDF cells. **d** Fluorescence micrographs of dead green stain in HDF cells in control, irradiated and pMMP-2sh transfected irradiated HDF cells. **e** Representative images γH2AX foci obtained by immunofluorescence microscopy in HDF cells in control, irradiated HDF cells and pMMP-2 transfected irradiated HDF cells. **f** Representative images of alkaline comet assay performed in HDF cells in control, irradiated and pMMP-2 transfected irradiated HDF cells

The cytotoxic effect of IR on the viability of HDFs was determined by MTT assay. The results show that the viability of HDFs was decreased with an increased dose of IR (2-10Gy) at 24, 48 and 72 h. The viability of HDFs was found to be 65 ± 3.4, 51 ± 3.9, 45 ± 4.8% at 24, 48 and 72 h with 10Gy radiation dose (Fig. 1a). After determining the dose-response from 2 to 10Gy, the effect of single dose radiation with 10Gy at 48 h was used to evaluate the effect of pMMP-2 on IR induced cytotoxicity in HDFs. Results indicate that the viability of irradiated HDFs with pMMP-2 transfection was increased 91 ± 4.0% compared to irradiated HDFs (Fig. 1b). The cytological changes in pMMP-2 transfected irradiated HDF cells at 10Gy were observed under phase contrast microscopy. As compared to non-irradiated control, irradiated HDFs showed morphological changes including the disrupted cell membrane with characteristics of apoptosis. However, pMMP-2 transfection apparently decreased the characteristics of apoptosis in irradiated HDFs (Fig. 1c). Further, the effect of pMMP-2 on IR induced cell death was evaluated using fluorescence-based dead green viability staining method in HDFs. The results display that fluorescence intensity of dead green viability stain was increased in irradiated HDFs compared to non-irradiated HDFs. However, pMMP-2 has decreased the fluorescence intensity of dead green viability stain in irradiated HDFs indicating the increased viability of irradiated HDFs. Mean fluorescence intensity (MFI) analysis by using Image J indicate that percent of HDF cells death with IR was 62.5 ± 4.5% compared to non-irradiated control (8.9 + 2.5%).The viability of pMMP-2 treated irradiated HDFs was 12.45 ± 3.9% after 48 h of post-irradiation (Fig. 1d). This study further extended to evaluate the effect of pMMP-2 transfection on radiation-induced DNA damage in HDF cells using γH2AX antibody. The results show that γH2AX foci were significantly increased with 10Gy radiation dosage at 48 h compared to non-irradiated control. Further, pMMP-2 has decreased γH2AX foci in irradiated HDFs. The analysis of foci by Image J displayed 8 ± 4.2, 75 ± 6.6 and 14 ± 4.5% of γH2AX foci in non-irradiated, irradiated and pMMP-2 transfected irradiated HDFs (10Gy) at 48 h, respectively (Fig. 1e). The effect of IR on DNA fragmentation was determined by the alkaline comet assay and the tail length was measured by using CASP Lab software. The results demonstrate that the tail length was increased about 20-folds with 10Gy radiation compared to non-irradiated control, whereas silencing of the MMP-2 gene has decreased the comet length to 4-folds in irradiated HDFs (Fig. 1f).

### Effect of MMP-2 gene silencing on IR induced DNA damage in MCF-7 cells

**Fig. 2:**
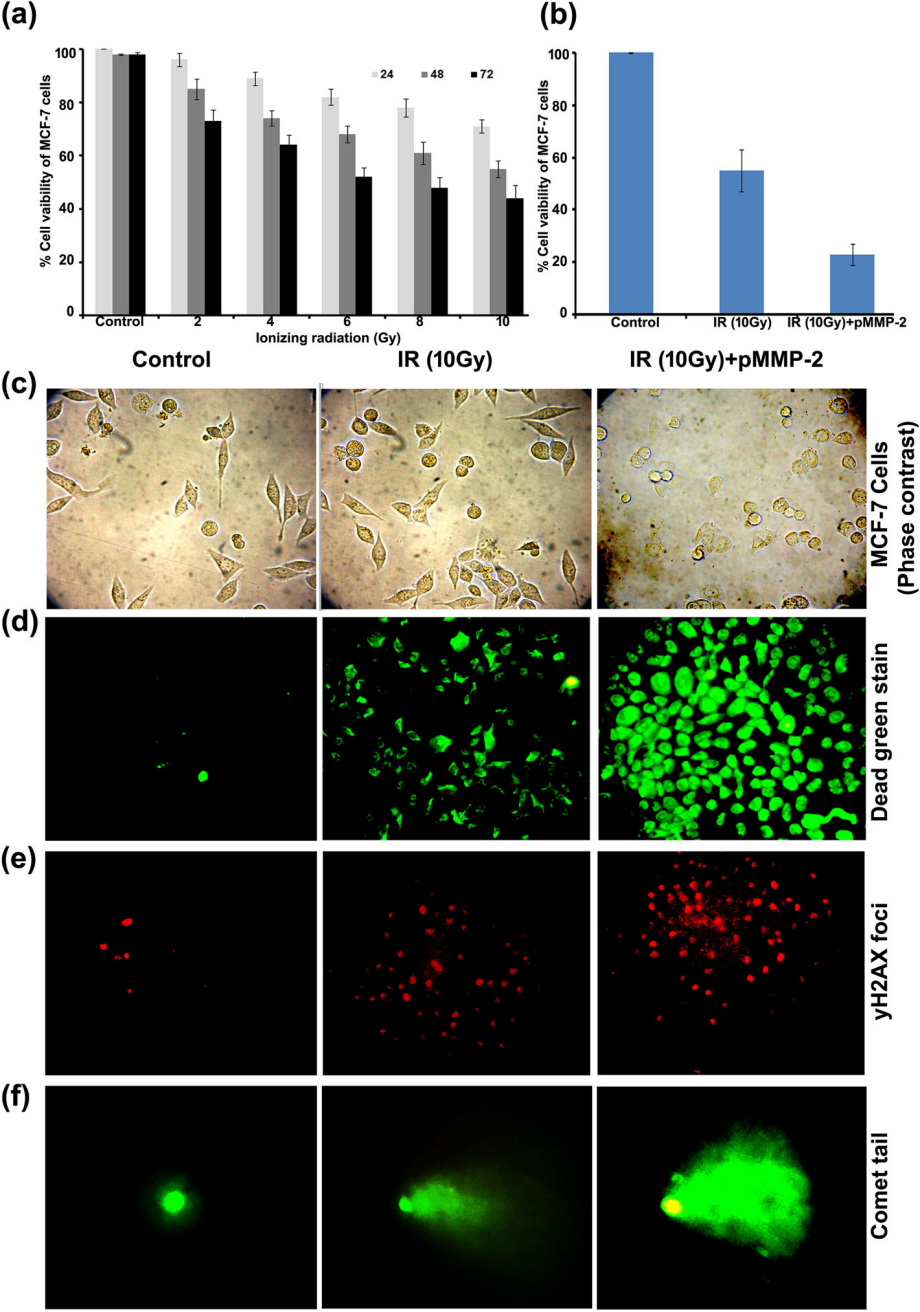
Effect of MMP-2 gene silencing in pMMP-2 transfected irradiated MCF-7 cells. **a** Graphical representation of viability of MCF-7 cells radiation (2-10Gy) analyzed by MTT assay. **b** Graphical representation of viability of pMMP-2 transfected MCF-7 cells at single large dose i.e. 10Gy analyzed by MTT assay. **c** Morphological characterization under phase contrast microscopy of MCF-7 cells. **d** Fluorescence micrographs of dead green stain in MCF-7 cells in control, irradiated and pMMP-2 transfected irradiated MCF-7 cells. **e** Representative images γH2AX foci obtained by immunofluorescence microscopy in MCF-7 cells in control, irradiated MCF-7 cells and pMMP-2 transfected irradiated MCF-7 cells. **f** Representative images of alkaline comet assay performed in MCF-7 cells in control, irradiated and pMMP-2 transfected irradiated MCF-7 cells

The viability of MCF-7 cells was decreased in a dose-dependent manner at 24, 48 and 72 h. The viability of MCF-7 cells was 71 ± 2.5, 55 ± 3.2 and 44 ± 4.8% at 24, 48 and 72 h with 10Gy radiation at 48 h (Fig. 2a). However, pMMP-2 transfection reduced the viability of irradiated MCF-7 cells by 23 ± 2.09% at 48 h (Fig. 2b). Morphological characteristics of apoptosis were featured with round shape, cell blebbing and shrinkage were increased with pMMP-2 transfection in irradiated MCF-7 cells (Fig. 2c). Further, the effect of pMMP-2 on IR induced cell death of MCF-7 by dead green assay showed that MFI of irradiated MCF-7 was 58 ± 8.9% and pMMP-2 transfected irradiated MCF-7 was 89 ± 4.6% (Fig. 2d). pMMP-2 also showed DNA damage effect more effectively in pMMP-2 transfected irradiated MCF-7 cells, γH2AX foci analysis by Image J indicated that the percentof foci was 69 ± 9.4% in irradiated MCF-7 cells and 9 ± 7.8% in non-irradiated MCF-7 cells, whereas 91 ± 5.6% in pMMP-2 transfected irradiated MCF-7 cells (Fig. 2e). DNA fragmentation analyses by comet assay indicate that the comet tail length was increased 7-folds in irradiated MCF-7 cells compared to non-irradiated control. However, pMMP-2 transfection further increased the tail length by 1.4 folds in irradiated MCF-7 (Fig. 2f).

## Discussion

Radiotherapy is always on a sharp edge in killing the tumors and limiting the survival of critically important adjacent normal tissues. Currently available radioprotective agents are non-specific and not fulfilling the criteria that have been approved for clinical use. Therefore, the development of target-specific radioprotective agents that exclusively protect normal cells but damage cancer cells is a choice of interest. The present study observed that dose-response versus viability was decreased from 2-10Gy IR at 24, 48 and 72 h in both HDFs and MCF-7 cells indicating the cytotoxic effect of IR. Previously, similar results were reported in murine skin fibroblasts [21], HEM normal human cells [22] and primary lung fibroblasts [23]. Further, 50% viability was observed with a single dose of IR (10Gy) at 48 h of post-radiation. Hence, the effect of MMP-2 gene silencing on the viability of HDFs and MCF-7 cells with IR (10Gy) at 48 h was evaluated similarly to an earlier study [24].

Loss of cell viability of irradiated HDFs and MCF-7 cells is due to DNA damage as detected by immunofluorescence method using γH2AX antibody. The H2AX is quickly phosphorylated at serine 139 when DSBs arise in the cells and form distinct γH2AX foci, an indicator of DNA damage. It plays a critical role in the recruitment of repair or damage-signaling factors at the site of DNA damage. Earlier, report on HF19 human fibroblasts observed an increased γH2AX foci with radiation [25]. This study demonstrated that the expression of γH2AX foci was decreased with pMMP-2 transfection in irradiated HDFs whereas increased in irradiated MCF-7 cells. Similarly, olive tail length due to DNA fragmentation decreased in irradiated HDFs and increased in MCF-7 cells. These results indicate DNA damage protection in HDFs and destruction in MCF-7 cells. Similarly, MnSOD siRNA showed a protective effect against oxidative stress and radiation exposure in mouse embryonic cells [26]. Glyburide, a small molecule inhibitor of protective genes effectively decreased radiation-induced cell death in T98G, U-87 MG, normal lung epithelial BEAS-2B and in primary astrocytes [27]. Whereas silencing of HIF-1 α gene protected the HepG2 cells from low doses of CoCl_2_ and radiation [28]. siRNA of pro-apoptotic genes, pkcδ and BAX reduced radiation-induced DNA damage in primary salivary gland cultures [18]. Silencing of Egr1 attenuated radiation-induced apoptosis in normal tissue, while sensitized the two cancer cell lines due to differential response based on the cellular context [29].

Ideally, clinically useful radioprotectors should protect normal cells and eliminate cancers. The differential response to DNA damage on the silencing of the MMP-2 gene in HDFs and MCF-7 cells might be due to the pro-death and pro-life activity of the pMMP-2. pMMP-2 has pro-death activity by causing DNA damage in HDFs and pro-life activity by inhibiting the DNA damage in MCF-7 cells. IR induced MMP-2 modulates ROS generation [30], which in turn causes DNA damage and cell death [31]. Silencing of MMP-2 gene reduced the DNA damage in HDFs, and protected cardiomyocyte from ischemia-reperfusion injury [32] and reduced the osteogenic transformation of fibroblasts by inhibiting the SMP/Smad pathway in ankylosing spondylitis [33]. The pro-death activity of the pMMP-2 enhanced DNA damage with IR in MCF-7 cells. Similarly, silencing of the MMP-2 gene, using MMP-2 siRNA transfection inhibited radiation-enhanced viability of glioma cells [34] and also suppressed the growth of esophageal carcinoma cells [35].However, pMMP-2 pro-death and pro-life activity may be due to ROS generation that promotes anti- or pro-tumor effects with different mechanisms and genomic instability [31]. Additional studies will be required to determine the differential mechanism in HDFs and MCF-7 cells.

## Conclusion

This study observed dose-dependent IR induced cytotoxicity in HDFs and MCF-7 cells with 50% reduction of viability by 10Gy at 48 h. Silencing of MMP-2 using pMMP-2 reduced the IR induced cytotoxicity and DNA damage and increased the viability of HDFs. However, MMP-2 gene silencing increased the cytotoxicity and DNA damage and decreased the viability of MCF-7 cells. This opposite effect of MMP-2 silencing in HDFs and MCF-7 cells might be due to the differential regulation of ROS generation.

## Additional file

Additional file 1: **Figure S1.** Expression of MMP-2 gene on silencing with MMP-2sh vector. a Western blot analysis of MMP-2 protein in HDFs transfected with pMMP-2. b Western blot analysis of MMP-2 protein in MCF-7 cells transfected with pMMP-2. (JPG 245 kb)

## Data Availability

Not applicable.

## Abbreviations

ECM: Extracellular matrix
HDF: Human normal Dermal Fibroblasts
IR: Ionizing radiation,
MFI: Mean Fluorescence Intensity
MMP: Matrix metalloproteinases
pMMP-2: Plasmid MMP-2 shRNA
siRNA: small interfering RNA
