# Supplementary figures and images for: Effect of MMP-2 gene silencing on radiation-induced DNA damage in human normal dermal fibroblasts and breast cancer cells
Source: Genes Environ. 2019 Jul 22;41:16. doi: 10.1186/s41021-019-0131-x (PMC6647068; doi:10.1186/s41021-019-0131-x)

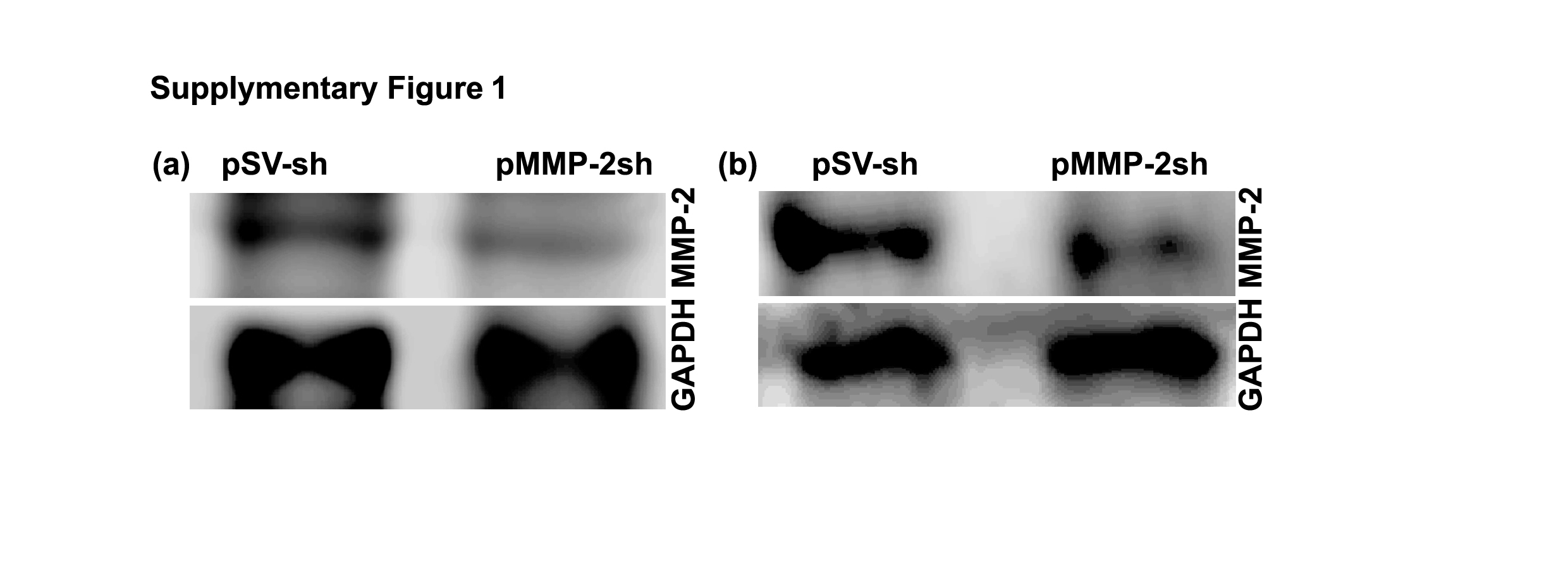

Supplement: Supplementary file 1 — Figure S1. Expression of MMP-2 gene on silencing with MMP-2sh vector. a Western blot analysis of MMP-2 protein in HDFs transfected with pMMP-2. b Western blot analysis of MMP-2 protein in MCF-7 cells transfected with pMMP-2. (JPG 245 kb) [file 41021_2019_131_MOESM1_ESM.jpg]
